# Extreme Enlargement of the Inverted Repeat Region in the Plastid Genomes of Diatoms from the Genus *Climaconeis*

**DOI:** 10.3390/ijms22137155

**Published:** 2021-07-02

**Authors:** Romain Gastineau, Nikolaï A. Davidovich, Olga I. Davidovich, Claude Lemieux, Monique Turmel, Rafał J. Wróbel, Andrzej Witkowski

**Affiliations:** 1 Institute of Marine and Environmental Sciences, University of Szczecin, Mickiewicza 16a, 70-383 Szczecin, Poland; nickolaid@mail.ru (N.A.D.); andrzej.witkowski@usz.edu.pl (A.W.); 2 Karadag Scientific Station–Natural Reserve of the Russian Academy of Sciences, p/o Kurortnoe, Feodosiya, 98188 Crimea, Russia; olivdav@mail.ru; 3 Département de Biochimie, de Microbiologie et de Bio-Informatique, Institut de Biologie Intégrative et des Systèmes, Université Laval, Québec, QC G1V 0A6, Canada; claude.lemieux@bcm.ulaval.ca (C.L.); monique.turmel@bcm.ulaval.ca (M.T.); 4 Engineering of Catalytic and Sorbent Materials Department, Faculty of Chemical Technology and Engineering, West Pomeranian University of Technology Szczecin, Pułaskiego 10, 70-322 Szczecin, Poland; rafal.wrobel@zut.edu.pl

**Keywords:** diatoms, *Climaconeis*, plastid genome, plastome, inverted repeat expansion, cryptic diversity

## Abstract

We sequenced the plastid genomes of three diatoms from the genus *Climaconeis*, including two strains formerly designated as *Climaconeis scalaris*. At 208,097 and 216,580 bp, the plastid genomes of the latter strains are the largest ever sequenced among diatoms and their increased size is explained by the massive expansion of the inverted repeat region. Important rearrangements of gene order were identified among the two populations of *Climaconeis* cf. *scalaris*. The other sequenced *Climaconeis* chloroplast genome is 1.5 times smaller compared with those of the *Climaconeis* cf. *scalaris* strains and it features an usual quadripartite structure. The extensive structural changes reported here for the genus *Climaconeis* are compared with those previously observed for other algae and plants displaying large plastid genomes.

## 1. Introduction

Diatoms represent a group of photosynthetic stramenopiles that are surrounded by a shell (frustule) made of silica. These algae play an important role in the carbon and silica cycles of the oceans as well as in primary production and the food chain. They are also good bioindicators, and can be used in many various fields, from biotechnology to forensic sciences. Fossil diatoms are important in oceanology and are used to reconstruct paleoclimates. More than 1200 genera of diatoms are known, with new species and genera being regularly discovered and described [1].

In general, the identification of diatoms requires observations in light (LM) and scanning electron microscopes (SEM). LM will provide general information about the outline and shape. It can be completed by SEM, which allows investigation of smaller details such as the shape and density of areolae or the presence/absence of processes such as rimoportulae and fultoportulae [1]. However, these tools are sometimes not sufficient to distinguish between cryptic species. Indeed, cryptic species are hard or impossible to distinguish based only on their morphologies. They might be biological species, reproductively isolated from morphologically similar species, also showing genetic differences when compared by the means of molecular barcoding [2,3,4,5,6,7,8].

*Climaconeis* Grunow 1862 is a small genus of pennate, raphid diatoms, with elongated valves [1,9]. The type species for this genus is *Climaconeis lorenzii* Grunow [9,10]. Currently, Algaebase [11] lists 22 species, with 18 taxonomically accepted. Among them, the species *Climaconeis scalaris* Brébisson E.J. Cox [12] is considered to be cosmopolitan, having been observed in several distant places. The type material, previously described as *Frustulia scalaris*, comes from Calvados, a French Department in Normandy, located near the English Channel [13]. It was also observed in Honduras as *Berkeleya scalaris* [9] and in the Black Sea as *Okedenia scopulorum* [14]. 

Recently, Davidovich et al. [15] described for the first time the complete process of sexual reproduction (also known as auxosporulation) of *C. scalaris*. The biological material used for this study originated from the Black Sea, where *C*. *scalaris* was previously observed by Mereschkowsky [14]. Subsequently, using an identical protocol, mating experiments were performed between clonal cultures of the Black Sea population and clones identified as *C. scalaris* from the Canary Islands (Davidovich, personal communication). All these mating experiments were unsuccessful, without any sexual stages being ever observed (e.g., formation of gametes and zygotes, development of an auxospore). However, the absence of positive results is not sufficient to conclude that the two populations of diatoms are reproductively isolated. This is because auxosporulation is regulated by many factors, biotic as well as abiotic, which may differ between species [16]. In addition, it is important to point out here that geographical separation of diatoms is not always linked with reproductive isolation. Indeed, a previous study conducted on the blue diatom *Haslea ostrearia* (Gaillon) Simonsen 1974 has shown that two distant populations from the Atlantic Coast of France and the Swedish side of the Kattegat strait, distinguishable by their mitochondrial genes, were able to interbreed and give birth to a viable progeny [17]. On the other hand, sympatric cryptic species of *Pseudo-nitzschia* spp. H. Peragallo 1900 have been shown to be reproductively isolated [2]. 

Considering the geographical distance between populations of *Climaconeis* cf. *scalaris* from the Black Sea and the Canary Islands, it is also possible that they represent cryptic species. To investigate this possibility, we sequenced the complete plastid genomes (cpDNA) of representatives from these two populations (i.e., the SZCZ 1888 strain originating from the Black Sea and the SZCZ 1889 strain from the Canary Islands) plus the plastid genome of an unidentified species of *Climaconeis* from New Zealand (SZCZ 1890). Our results revealed that the two *Climaconeis* cf. *scalaris* strains exhibit important genetic differences, which qualify them as distinct cryptic species. Unexpectedly, we found that the quadripartite structure of their cpDNA differs extensively from the typical organization observed among all previously examined diatoms and the vast majority of photosynthetic organisms. 

## 2. Results

### 2.1. Morphology

**Figure 1:**
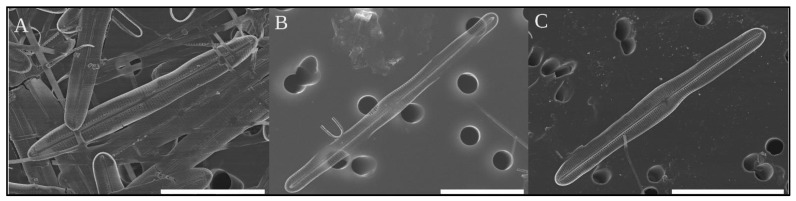
(**A**) SEM image of cleaned frustules of *Climaconeis* cf. *scalaris* SZCZ 1888, scale bar = 20 μm. (**B**) SEM image of a cleaned frustule of *Climaconeis* cf. *scalaris* SZCZ 1889, scale bar = 20 μm. (**C**) SEM image of a cleaned frustule of *Climaconeis* sp. SZCZ 1890, scale bar = 30 μm.

SEM pictures of the three clones used in this study are shown in Figure 1A–C. Measurements of length, width, and density of areolae are reported in Table 1.

Based on the morphometrics gathered from Figure 1, it is possible to ascribe SZCZ 1888 and SZCZ 1889 to *Climaconeis* cf. *scalaris*. The only slight difference observed between both strains is the density of transapical stria. SZCZ 1890 displays more differences in its density of longitudinal stria.

### 2.2. Structure of the Sequenced Plastid Genomes

The plastid genomes of all three *Climaconeis* spp. clones contain two copies of a large inverted repeat (IR) that are separated from one another by large single-copy (LSC) and small single-copy (SSC) regions. The gene content of these different regions is presented in Table 2.

**Figure 2:**
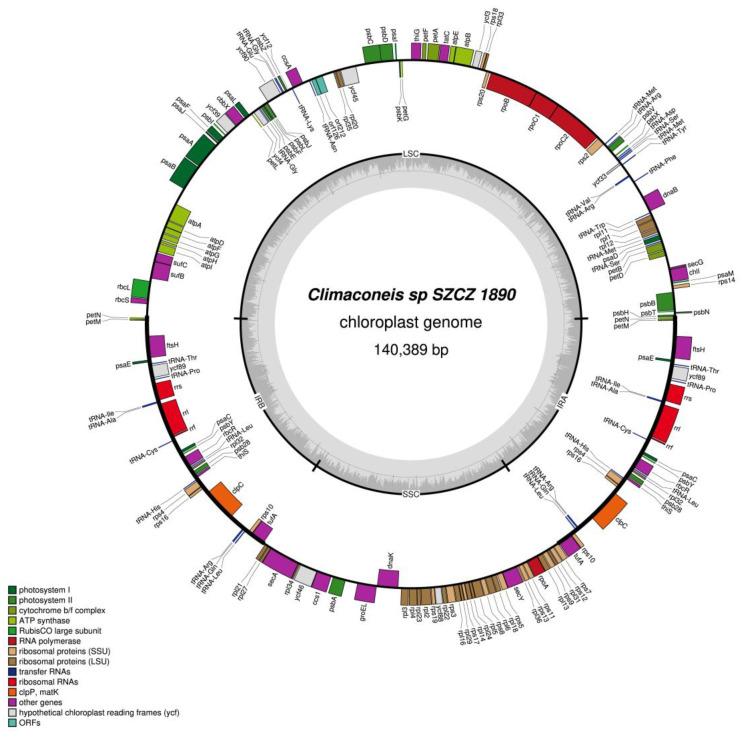
Map of the plastid genome of *Climaconeis* sp. SZCZ 1890.

The plastid genome of *Climaconeis* sp. SZCZ 1890 is 140,389 bp long (GenBank: MZ365053) and possesses the typical quadripartite structure that has been observed for other diatom cpDNAs (Figure 2), except that the 22,657 bp IR is similar in size to the SSC region (26,303 bp). The IR contains 16 protein-coding genes, 3 rRNA genes, and 10 tRNA genes. Located at one of its endpoints are the *tufA* and *rps10* genes, which are usually part of a large conserved cluster of ribosomal protein genes. Note that on one of the IR copies (IRB), the 3′ end of *tufA* is positioned next to the 5′ end of *rps7* (a gene present in the SSC region), thus reconstituting the abovementioned ribosomal protein cluster. Table 3 compares the gene content of the IR of *Climaconeis* sp. SZCZ 1890 with those of four other members of the order Naviculales. As it can be seen, the IR of *Climaconeis* sp. SZCZ 1890 features many extra genes. The common denominator of all IRs from Naviculales is the seven-gene cluster comprising *tRNA-Pro, ycf89, rns, tRNA-Ile, tRNA-Ala, rnl, and rrf,* in which non-conserved ORFs are sometimes interspersed. In the case of *Climaconeis* sp. SZCZ 1890, this cluster also exists, but the positions of *ycf89* and *tRNA-Pro* are reverse. The SSC encodes 37 protein-coding genes, most of which form the large cluster of ribosomal protein genes. Genes in the SSC region are tightly packed, with non-coding sequences mostly restricted to the 1040 bp spacer between *psbA* and *groEL*. On the other hand, the LSC region of *Climaconeis* sp. SZCZ 1890 is 68,771 bp long and encodes 68 protein-coding genes, 16 tRNA genes and two non-conserved ORFs (*orf212* and *orf126*).

In contrast, the plastid genome organization of *Climaconeis* cf. *scalaris* SZCZ 1888 (GenBank: MZ365054) and SZCZ 1889 (GenBank: MZ365055) differs remarkably from that of *Climaconeis* sp. SZCZ 1890. In the former strains, the IR is at least 3.4-fold longer (longer than the LSC region) and contains at least 15 additional genes, whereas the SSC region is a reduced sequence of only 300–400 bp carrying no genes. This situation is also reflected by the substantial increase in size of these genomes, which exceeds 200 kb.

**Figure 3:**
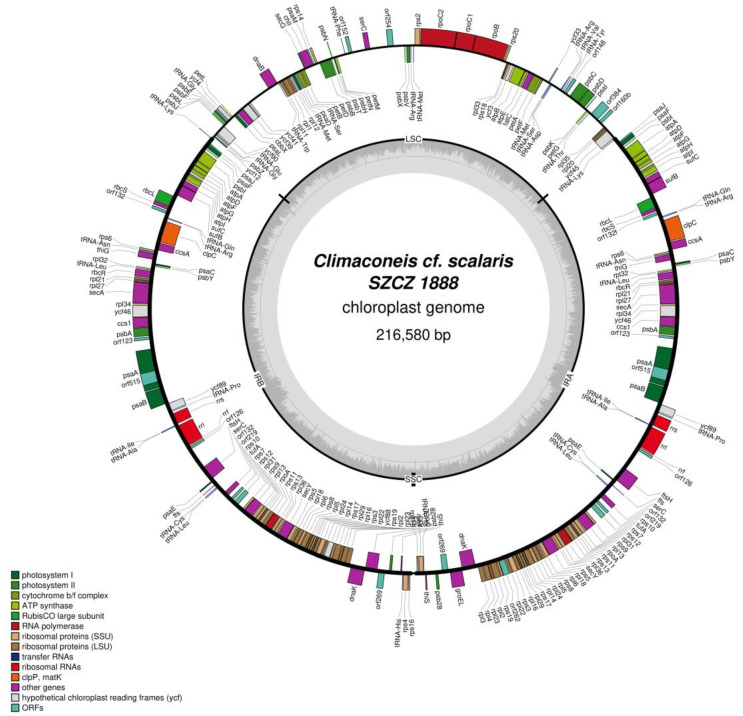
Map of the plastid genome of *Climaconeis* cf. *scalaris* SZCZ 1888.

The 216,580 bp plastid genome of *Climaconeis* cf. *scalaris* SZCZ 1888 possesses an IR of 78,313 bp that encodes 69 conserved protein-coding genes, a putative *serC* gene, 6 non-conserved ORFs, and 15 RNA genes coding for 3 rRNAs, 11 tRNAs, and the RNA component (ffs) of the signal recognition particle (SRP) ribonucleoprotein complex (Figure 3). The LSC region is 59,560 bp long and encodes 54 conserved protein-coding genes, 5 non-conserved ORFs, a putative serine recombinase *serC* gene, and 16 tRNA genes. The SSC is a non-coding sequence of 394 bp. The two putative *serC* genes are intact and not pseudogenes [18].The genome contains a single intron, a group II intron in *psaA* with a 515 amino-acid long ORF coding for a putative reverse transcriptase. Similar introns in the same gene have been previously identified in various microalgae such as the diatoms *Toxarium undulatum* Bailey 1854 (AOS86555) [16] and *Haslea silbo* Gastineau, Hansen and Mouget 2021 (QUS63628 and QUS63831) [19], the silicoflagellate *Dictyocha speculum* Ehrenberg 1839 (QDH81707) [20], the red alga *Bulboplastis apyrenoidosa* A.Kushibiki, A.Yokoyama, M.Iwataki, J.Yokoyama, J.A.West & Y.Hara 2012 (ARO90857) [21], and the green alga *Chlamydomonas applanata* Pringsheim 1930 (ALO63257) [22]. The best blastp hit (62.02% identity and E-value = 0.0) was obtained with the *D. speculum* intron-encoded protein.

**Figure 4:**
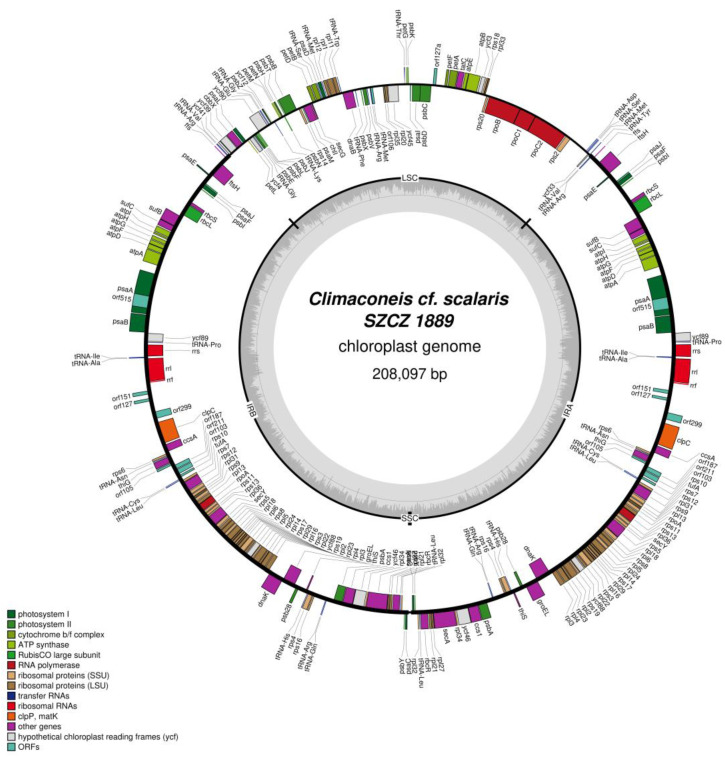
Map of the plastid genome of *Climaconeis* cf. *scalaris* SZCZ 1889.

The plastid genome of *Climaconeis* cf. *scalaris* SZCZ 1889 is 208,097 bp long (Figure 4). Its IR of 79,040 bp contains 69 conserved protein-coding genes, 7 non-conserved ORFs, and 16 RNA genes coding for 3 rRNAs, 12 tRNAs, and the RNA component (ffs) of the signal recognition particle (SRP) ribonucleoprotein complex. The LSC region of 49,713 bp encodes 54 conserved protein-coding genes, 2 non-conserved ORFs, and 14 tRNA genes. The gene-less SSC region is 304 bp long. A group II intron encoding a putative reverse transcriptase of 515 amino-acid is present in *psaA*; however, this protein sequence shows only 77.67% identity with the *psaA* intron-encoded protein of the SZCZ 1888 strain.

**Figure 5:**
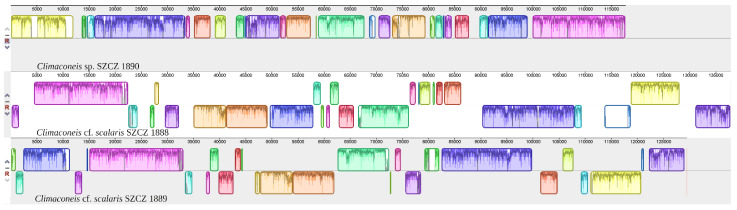
MAUVE alignment of the plastid genomes of *Climaconeis* sp. SZCZ 1890, *Climaconeis* cf. *scalaris* SZCZ 1888, and *Climaconeis* cf. *scalaris* SZCZ 1889, using as reference the *Climaconeis* sp. SZCZ 1890.

Comparison of gene order of the three sequenced plastid genomes indicated that the *Climaconeis* sp. SZCZ 1890 genome is extensively rearranged relative to those of the *Climaconeis* cf. *scalaris* strains (Figure 5).

**Figure 6:**
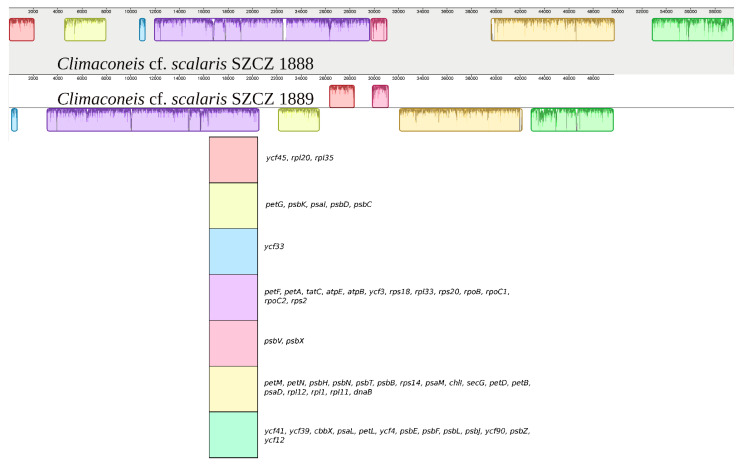
MAUVE alignment of the LSC regions from *Climaconeis* cf. *scalaris* SZCZ 1888 and *Climaconeis* cf. *scalaris* SZCZ 1889. The legend below shows the gene content of the blocks of synteny (conserved protein-coding genes only).

**Figure 7:**
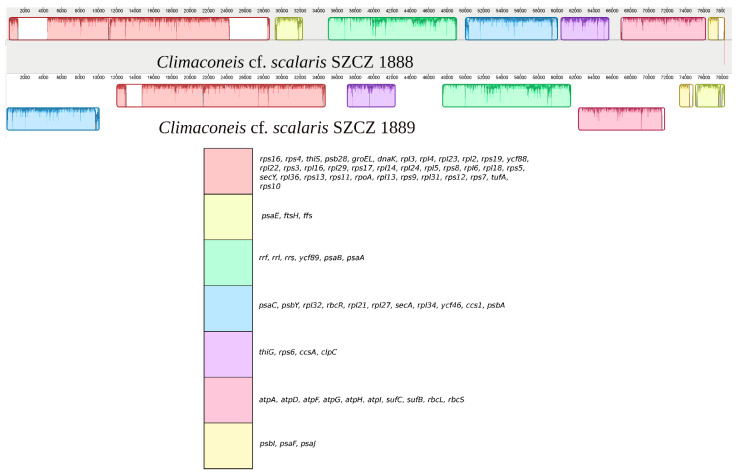
MAUVE alignment of the IR regions from *Climaconeis* cf. *scalaris* SZCZ 1888 and *Climaconeis* cf. *scalaris* SZCZ 1889. The legend shows the gene content of the blocks of synteny (conserved protein-coding genes and rRNA only).

Moreover, a number of differences in gene order were identified between the SZCZ1888 and SZCZ1889 genomes when their protein-coding and rRNA genes were examined (Figure 6 for the LSC and Figure 7 for the IR). A total of 125 genes were associated with 14 syntenic blocks containing 1 to 36 genes that conserved protein-coding genes. Note here that these two genomes are identical in gene content with regards to protein and RNA-coding genes. The case of *ycf33* is slightly different, as it is the only protein-coding gene of the block, but is always associated with *tRNA-Asp*, *tRNA-Ser,* and *tRNA-Met* among the three clones sequenced.

## 3. Discussion

Our study shed light on the biodiversity of the genus *Climaconeis* by providing evidence for the existence of cryptic species. Using microscopy alone, it was difficult to differentiate between the three examined strains, especially between *Climaconeis* cf. *scalaris* SZCZ 1888 and SZCZ 1889, as they differ slightly in length and width (Table 1) and these differences disappear progressively during the life cycle because of the MacDonald-Pfitzer rule. SZCZ 1888 and 1889 clearly belong to *C. scalaris* on the basis of their morphological characteristics [12,23]; however, without the molecular data derived from the plastid genome, it would have been difficult to distinguish them. Even an analysis based on a very broadly used molecular barcode marker such as the small subunit of the nuclear ribosomal RNA gene (SSU or 18S) would not have been so conclusive, considering that the complete SSU genes of SZCZ 1888 (MZ365054) and SZCZ 1889 (MZ365055) share 99.49% identity, with eight out of the nine polymorphisms detected mapping to the first 700 bp of the 1769 bp sequence. Indeed, molecular barcoding, which is typically based on analysis of PCR-amplified genes, might have been unable to discriminate between the two strains depending on the amplification primers used and the size of the fragments generated. In this context, note here that the plastid-encoded *rbcL* gene, another marker widely used for barcoding, would allow one to discriminate more efficiently the SZCZ 1888 and 1889 strains, given that their *rbcL* genes show 97.48% identity.

The most unexpected finding of our study is the great variability of the *Climaconeis* plastid genome at both the size and gene organization levels. At 216,580 and 208,097 bp, respectively, the cpDNAs of the SZCZ 1888 and SZCZ 1889 strains are 1.5 times larger than the cpDNA of SZCZ 1890 (140,389 bp), representing the largest known plastid genomes among diatoms. Their increased sizes are accounted for by the 3.5-fold expansion of the IR (from about 23 kb to 79 kb) into neighboring single-copy regions, an event that was accompanied with the acquisition of all the genes from the SSC region together with the extreme reduction of the latter region to a non-coding region of less than 400 bp. Prior to our study, the largest recorded diatom cpDNA was the 201,816 bp plastome of *Plagiogramma staurophorum* (MG755792), formerly known to harbor the largest known IR (34,888 bp) among diatoms [24]; however, the extra length of this IR is mainly due to enlargement of intergenic spacers, rather than gains of multiple genes through the invasion of the IR into single-copy regions. Variation in gene content of the IR has been previously documented for other diatoms, for instance for *Eunotia naegelii* (KF733443) and the centric species *Toxarium undulatum* (KX619437), whose IRs are 27,185 bp and 19,642 bp long, respectively [18,25]. Although change in IR length is generally the major source of plastid genome size variation among diatoms, gains of sequences through horizontal transfers or recombination with plasmids sometimes contribute to larger genome size. This is the case for the 156,157 bp cpDNA of *H. silbo* that acquired a sequence of about 30 kb in the LSC region through recombination with plasmids [19].

The cpDNA architecture reported here for *Climaconeis* cf. *scalaris* SZCZ 1888 and SZCZ 1889 is comparable to those observed for the green algae *Scherffelia dubia* (Perty) Pascher 1912 (KU167098) and *Tetraselmis* sp. (KU167097) [26], which are both members of the Chlorodendrophyceae (Chlorophyta). Despite their substantially smaller sizes (137,161 bp for *S. dubia* and 100,264 bp for *Tetraselmis* sp.) relative to their *Climaconeis* spp. counterparts, these green algal cpDNAs also feature long, gene-rich IRs (32,310 bp and 21,342 bp, respectively) and a highly reduced SSC region consisting of a non-coding sequence (392 bp for *Tetraselmis* sp. and 3385 bp for *S. dubia*). This type of architecture appears to be conserved in the genus *Tetraselmis* F. Stein, 1878, as recent sequencing of the 149,934 bp cpDNA of *Tetraselmis desikacharyi* Marin, Hoef-Emden and Melkonian 1996 disclosed a 38,343 bp IR and a 1611 bp SSC [27] (sequence available on the CNGB Nucleotide Sequence Archive under accession number CNP0000390).

The plastid genomes of other species of Ochrophyta are also known for their long, gene-rich IR and for their short SSC sometimes encoding a few genes [28,29,30]. For example, the 126,746 bp cpDNA of the Chrysophyceae *Ochromonas* sp. (KJ877675) [30] contains a 22,906 bp IR and a 805 bp SSC encoding two protein-coding genes. Furthermore, among Ochrophyta, the Synurophyceae *Neotessella volvocina* (Playfair) B.Y. Jo, J. I. Kim, W. Shin, P. Skaloud, and P. Siver 2016 has a 130,705 bp cpDNA with 24,064 bp IR and a 2432 SSC devoid of any genes (MH795132) [29].

Considering that land plant plastid genomes have a typical IR size of 25–30 kb, the genus *Pelargonium* L’Hér., 1789 (Geraniaceae), is exceptional in boasting the largest plastid genomes with IRs ranging up to 88 kb [31]. This genus, which contains four subgenera, represents a good model system to analyze large-scale expansions of the IR and the evolution of plastome structure in general. A comparison of 22 *Pelargonium* species allowed the identification of 10 distinct genome organizations (type I-X) based on inversions and IR/LSC boundary variation [31]. The reconstructed evolutionary scenario disclosed 7 separate events of IR expansion, 2 events of IR contraction, and 13 inversions. Only two species, *Pelargonium transvaalense* Knuth (KM527900), and *Pelargonium endlicherianum* Fenzl (KX267771) displayed an IR longer than those of *Climaconeis* cf. *scalaris* SZCZ 1888 and SZCZ 1889 that also contains the entire sequence of the large ribosomal protein operon (this is not the case for the Chlorodendrophyceae, Chrysophyceae, and Synurophyceae). The SSC regions of all *Pelargonium* species shared a few protein-coding genes.

The major changes in IR size and gene content as well as the numerous gene rearrangements we have identified in the present study suggest that, as is the case for *Pelargonium*, the plastid genome is evolving in a highly dynamic fashion in *Climaconeis*. Therefore, it is predicted that analyses of additional strains/populations from the *C. scalaris* species complex will uncover other important differences in cpDNA organization. Moreover, if these analyses are linked with studies on the interbreeding capacities of the investigated strains/populations, they are also likely to lead to the discovery of several cryptic species.

## 4. Materials and Methods

### 4.1. Biological Material

The strains of *Climaconeis* spp. examined in this study were assigned two accession numbers, one from the Karadag Scientific Station, where the strains were isolated and living cultures are being kept, and one from the Szczecin Diatom Culture Collection (SZCZ), where permanent slides and SEM stubs are conserved and where the molecular part of the study also took place. Note that, considering the outcome of our study, we use here the term Climaconeis cf. scalaris rather than C. scalaris. *Climaconeis* cf. *scalaris* 50716-A was isolated from the Black Sea, at Kuzmichevy kamni (44°54′40″ N, 35°12′46″ E) and was registered as SZCZ 1888. *Climaconeis* cf. *scalaris* 80601-A was isolated from the shores of Tenerife, Canary Islands (28°19′44″ N 16°21′46″ W) and registered as SZCZ 1889. A third strain of unidentified *Climaconeis* sp. 50703-G was isolated from Auckland, New Zealand (36°50′28″ S, 174°45′33″ E) and registered as SZCZ 1890. For the sake of concision, only the SZCZ accession numbers are provided in the other sections of this article. All three strains were grown at 21 °C under 14 h light/10 h darkness in F/2 medium [32] with a salinity of 35 PSU, except that the SZCZ 1888 strain was cultivated in 15 PSU medium. Illumination under an irradiance of ca. 80 μmol photons m^−2^ s^−1^ was provided by fluorescent tubes.

### 4.2. Scanning Electron Microscopy

An aliquot of approximately 1.5 mL of culture was transferred to an Eppendorf tube and cells were harvested by centrifugation. Subsequently, frustules were cleaned as follows; first they were boiled in HCl at 95 °C for 1 h, then washed with distilled water, boiled again in hydrogen peroxide at 95 °C for 1 h and finally rinsed at least 3 times with distilled water. Cleaned frustules were placed to dry on SEM stubs, before being coated with gold using a Q150T coater from Quorum Technologies (Laughton, U.K.). Observations were carried out using a SU8020 ultra-high resolution scanning microscope from Hitachi (Tokyo, Japan).

### 4.3. DNA Extraction, Sequencing and Analyses

Cultures were harvested during exponential growth phase by gentle centrifugation and total cellular DNA was extracted following the protocol of Doyle and Doyle [33]. For each strain, about 60 millions of 100 bp paired-end reads were generated by the Beijing Genomics Institute (Shenzhen, China) on the DNBSEQ platform. Reads were assembled using SPAdes 3.14.0 [34] with a k-mer of 85. Contigs corresponding to the cpDNA and nuclear-encoded 18S rRNA genes were retrieved by customized blastn analyses. The “addsolexareads” function of the Consed package [35] was used to circularize the plastid genome sequences. Genes were identified using the findORF script developed at Laval University [26,36]. Gene maps were drawn using the OGDRAW online platform [37]. Whole genome alignments were performed using the progressive alignment algorithm of Mauve [38], after eliminating one of the IR copies. The same software was used to align the individual IR and LSC regions from different strains. Identities between genes or protein sequences were calculated using Clustal Omega [39].

## 5. Conclusions

Our study demonstrated that the species *Climaconeis scalaris* encompass several cryptic species that are characterized by the large size of their plastid genome, the largest yet reported among diatoms. The increased sizes of these plastid genomes are mainly explained by the expansion of the IR to the detriment of the SSC region. The IR of *Climaconeis* sp. SZCZ 1890, which encodes only part of the genes making up the large ribosomal protein operon, appears to represent an intermediary state of expansion. Sequencing the plastid genomes of additional *Climaconeis* species would provide deeper insights into the dynamics of IR expansion in this genus.

## Figures and Tables

**Table 1:** Main morphological characteristics of the three clones of *Climaconeis* spp. sequenced in this study, as measured on the SEM images.

	Length (in μm)	Width (in μm)	Density of Transapical Stria in 10 μm	Density of Longitudinal Stria in 10 μm
*Climaconeis* cf. *scalaris* SZCZ 1888	47–53	5.2–7.0	19	28
*Climaconeis* cf. *scalaris* SZCZ 1889	62–66	5.0–6.2	20	28
*Climaconeis* sp. SZCZ 1890	47–64	6.2–6.8	20	24

**Table 2:** Genes identified in the different regions of the plastid genomes of the three clones of *Climaconeis* spp. sequenced in this study.

*Climaconeis* cf. *scalaris* SZCZ 1888	
LSC	*ycf45, rpl20, rpl35, orf160b, orf384, tRNA-Thr, petG, psbK, psaI, psbD, psbC, orf148, tRNA-Tyr, tRNA-Val, tRNA-Arg, tRNA-Asn, tRNA-Ser, tRNA-Met, ycf33, petF, petA, tatC, atpE, atpB, ycf3, rps18, rpl33, rps20, rpoB, rpoC1, rpoC2, rps2, tRNA-Met, tRNA-Arg, psbV, psbX, orf254, serC, orf152, tRNA-Phe, petM, petN, psbH, psbN, psbT, psbB, rps14, psaM, chlI, secG, petD, petB, tRNA-Ser, psaD, tRNA-Met, rpl12, rpl1, rpl11, tRNA-Trp, dnaB, ycf41, ycf39, cbbX, psaL, petL, ycf4, tRNA-Gly, psbE, psbF, psbL, psbJ, ycf90, tRNA-Glu, tRNA-Gly, psbZ, ycf12*
SSC	None
IR	*tRNA-Lys, psaJ, psaF, psbI, atpaA, atpD, atpF, atpG, atpH, atpI, sufC, sufB, rbcL, rbcS, orf132, tRNA-Gln, tRNA-Arg, clpC, ccsA, rps6, tRNA-Asn, thiG, psaC, psbY, rpl32, tRNA-Leu, rbcR, rpl21, rpl27, secA, rpl34, ycf46, ccs1, psbA, orf123, psaA + intron, psaB, ycf89, tRNA-Pro, rrs, tRNA-Ile, tRNA-Ala, rrl, rrf, orf126, ftsH, psaE, tRNA-Cys, tRNA-Leu, serC, orf132, orf219, rps10, tufA, rps7, rps12, rpl31, rps9, rpl13, rpoA, rps11, rps13, rpl36, secY, rps5, rpl18, rpl6, rps8, rpl5, rpl24, rpl14, rps17, rpl29, rpl16, rps3, rpl22, ycf88, rps19, rpl2, rpl23, rpl4, rpl3, dnaK, groEL, orf269, psb28, thiS, tRNA-His, rps4, rps16, ffs*
*Climaconeis* cf. *scalaris* SZCZ 1889	
LSC	*ycf33, tRNA-Met, tRNA-Ser, tRNA-Asp, rps2, rpoC2, rpoC1, rpoB, rps20, rpl33, rps18, ycf3, atpB, atpE, tatC, petA, petF, orf127a, psbC, psbD, psaI, psbK, petG, tRNA-Thr, ycf45, rpl20, rpl35, orf106, tRNA-Met, tRNA-Arg, psbV, psbX, tRNA-Phe, dnaB, tRNA-Trp, rpl11, rpl1, rpl12, tRNA-Met, psaD, tRNA-Ser, petB, petD, secG, chlI, psaM, rps14, psbB, psbT, psbN, psbH, petN, petM, tRNA-Lys, ycf12, psbZ, tRNA-Gly, tRNA-Glu, ycf90, psbJ, psbL, psbF, psbE, tRNA-Gly, ycf4, petL, psaL, cbbX, ycf39, ycf41*
SSC	None
IR	*tRNA-Val, tRNA-Arg, ffs, ftsH, psaE, psaJ, psaF, psbI, rbcS, rbcL, sufB, sufC, atpI, atpH, atpG, atpF, atpD, atpA, psaA + intron, psaB, ycf89, tRNA-Pro, rrs, tRNA-Ile, tRNA-Ala, rrl, rrf, orf151, orf127, orf299, clpC, ccsA, rps6, tRNA-Asn, thiG, orf105, orf187, orf211, orf103, tRNA-Cys, tRNA-Leu, rps10, tufA, rps7, rps12, rpl31, rps9, rpl13, rpoA, rps11, rps13, rpl36, secY, rps5, rpl18, rpl6, rps8, rpl5, rpl24, rpl14, rps17, rpl29, rpl16, rps3, rpl22, ycf88, rps19, rpl2, rpl23, rpl4, rpl3, dnaK, groEL, psb28, thiS, tRNA-His, rps4, rps16, tRNA-Arg, tRNA-Gln, psabA, ccs1, ycf46, rpl34, secA, rpl27, rpl21, rbcR, tRNA-Leu, rpl32, psbY, psaC*
*Climaconeis* sp. SZCZ 1890	
LSC	*psbH, psbN, psbT, psbB, rps14, psaM, chlI, secG, petD, petB, psaD, tRNA-Met, rpl12, rpl1, rpl11, tRNA-Trp, dnaB, tRNA-Phe, tRNA-Arg, tRNA-Val, tRNA-Tyr, ycf33, tRNA-Met, tRNA-Ser, tRNA-Asp, psbX, psbV, tRNA-Arg, tRNA-Met, rps2, rpoC2, rpoC1, rpoB, rps20, rpl33, rps18, ycf3, atpB, atpE, tatC, petA, petF, thiG, petG, psbK, psaI, psbD, psbC, ycf45, rpl20, rpl35, orf212, orf126, tRNA-Asn, ccsA, tRNA-Lys, ycf12, psbZ, tRNA-Gly, tRNA-Glu, ycf90, psbJ, psbL, psbF, psbE, tRNA-Gly, ycf4, petL, psaL, cbbX, ycf39, psbI, psaF, psaJ, psaA, psaB, atpA, atpD, atpF, atpG, atpH, atpI, sufC, sufB, rbcL, rbcS*
SSC	*rpl21, rpl27, secA, rpl34, ycf46, ccs1, psbA, groEL, dnaK, rpl3, rpl4, rpl23, rpl2, rps19, ycf88, rpl22, rps3, rpl16, rpl29, rps17, rpl14, rl24, rpl5, rps8, rpl6, rpl18, rps5, secY, rpl36, rps13, rps11, rpoA, rpl13, rps9, rpl31, rps12, rps7*
IR	*petN, petM, ftsH, psaE, tRNA-Thr, ycf89, tRNA-Pro, rrs, tRNA-Ile, tRNA-Ala, rrl, rrf, tRNA-Cys, psaC, psbY, rbcR, tRNA-Leu, rpl32, psb28, thisS, tRNA-His, rps4, rps16, clpC, tRNA-Arg, tRNA-Gln, tRNA-Leu, rps10, tufA*

**Table 3:** Comparison of the genes found in the IRs of *Climaconeis* sp. SZCZ 1890 and other members of the Naviculales. * refers to a gene identified in the course of this study but not annotated in the sequence reported in GenBank.

Species	Genes and ORFs in the Inverted Repeat
*Climaconeis* sp. SZCZ 1890	*petN, petM, ftsH, psaE, tRNA-Thr, ycf89, tRNA-Pro, rns, tRNA-Ile, tRNA-Ala, rnl, rrf, tRNA-Cys, psaC, psbY, rbcR, tRNA-Leu, rpl32, psb28, thisS, tRNA-His, rps4, rps16, clpC, tRNA-Arg, tRNA-Gln, tRNA-Leu, rps10, tufA*
*Seminavis robusta* MH356727	*tRNA-Pro, ycf89, rns, tRNA-Ile, tRNA-Ala, rnl, I-SroI* (endonuclease), rrf *
*Haslea nusantara* MH681881	*tRNA-Pro, ycf89, rns, tRNA-Ile, tRNA-Ala, rnl, rrf, psbY*
*Haslea silbo* MW645082, MW645084	*tRNA-Pro, ycf89, rns, tRNA-Ile, tRNA-Ala, rnl, rrf, psbY*
*Navicula veneta* MT383645	*orf161, tRNA-Pro, ycf89, orf140, orf154, rns, tRNA-Ile, tRNA-Ala, rnl, rrf, orf383*

## Data Availability

All molecular data have been deposited in GenBank.
